# Echoes of the Mind: Auditory Charles Bonnet Syndrome

**DOI:** 10.7759/cureus.66120

**Published:** 2024-08-04

**Authors:** Naresh D, Logesh D, Lakshmi Dorai B, Pradeep C, Hari Baskar S

**Affiliations:** 1 Psychiatry, Vinayaka Mission's Kirupananda Variyar Medical College and Hospitals, Salem, IND; 2 Psychiatry, Dhanalakshmi Srinivasan Medical College Hospital, Trichy, IND

**Keywords:** sensory deprivation, antipsychotic treatment, auditory charles bonnet syndrome, sensorineural hearing loss, musical hallucinations

## Abstract

Musical hallucinations (MH) represent a rare and complex auditory phenomenon where individuals perceive music without external stimuli. This case study explores auditory Charles Bonnet syndrome (ACBS) in a 51-year-old male with a history of bilateral sensorineural hearing loss. The patient reported hearing recognizable prayer chants, initially perceived as external sounds from a nearby temple. Over time, these hallucinations persisted and interfered with his daily activities, prompting medical consultation. Despite the absence of psychiatric illness, the patient was diagnosed with ACBS and treated with risperidone, an atypical antipsychotic. The intervention led to a significant reduction in the frequency and intensity of the hallucinations, alongside improved sleep and concentration. The patient also experienced a recurrence of symptoms upon discontinuation of the medication, highlighting the importance of adherence to treatment. This case underscores the need for awareness and understanding of non-psychotic auditory hallucinations in individuals with hearing impairments. The pathophysiology of MH is not fully understood but is believed to involve abnormal activity in the auditory associative cortices due to sensory deprivation. Treatment approaches often include both pharmacological and non-pharmacological strategies, such as optimizing hearing with aids and providing psychoeducation. This study contributes to the limited literature on ACBS and emphasizes the efficacy of antipsychotics in managing MH. Further research is essential to explore the underlying mechanisms and to develop comprehensive management plans for patients experiencing these distressing auditory phenomena. The findings advocate for a multidisciplinary approach to treatment, integrating audiological and psychiatric care to improve patient outcomes.

## Introduction

Musical hallucinations (MH) are a fascinating and complex phenomenon manifesting as the perception of music without any external auditory stimuli. These auditory hallucinations can be classified into two main categories: simple and complex. Simple hallucinations typically involve simpler sounds such as tinnitus or buzzing, while complex hallucinations encompass more elaborate sounds, including voices, speech, and music [1].

Auditory Charles Bonnet syndrome (ACBS), specifically refers to the occurrence of MH in individuals with sensorineural hearing loss. This condition was described by the renowned neurologist Oliver Sacks, who documented numerous cases of patients experiencing vivid MH despite the absence of psychiatric disorders. The phenomenon challenges our understanding of sensory perception and its underlying neural mechanisms, especially in the context of sensory deprivation [2].

Characteristics of musical hallucinations

MH in ACBS are distinct from other types of auditory hallucinations due to their complexity and the involvement of musical content. These hallucinations often consist of familiar tunes, songs, or even orchestral music. Patients with this condition typically retain their ability to differentiate these hallucinations from reality, maintaining intact reality testing. This differentiates ACBS from psychiatric conditions where hallucinations may be perceived as real [3].

MH often arise with significant hearing loss. Auditory deprivation can cause maladaptive neuroplastic changes in the auditory cortex, leading to spontaneous activity and the perception of music in the absence of external stimuli [4].

Neurobiological mechanisms

The neurobiological mechanisms of MH in ACBS are complex and not fully understood. They are thought to result from aberrant neural activity in the auditory cortex and related pathways. Functional imaging shows that brain areas involved in music perception, such as the auditory cortex, are activated during these hallucinations [5].

One hypothesis suggests that hearing loss leads to hyperactivity in the auditory cortex, similar to tinnitus mechanisms, as the brain compensates for the lack of input. Another theory posits that loss of auditory nerve input alters the balance of excitatory and inhibitory neurotransmission in the auditory cortex, resulting in MH [6].

MH in sensorineural hearing loss, seen in ACBS, highlight the brain's ability to create sensory experiences without external stimuli. Understanding its mechanisms provides insights into sensory processing and neuroplasticity, guiding effective treatments. Further research is needed to uncover the neural circuits involved and develop targeted therapies for this rare condition.

## Case presentation

A 51-year-old male power loom worker with a 30-year career and a history of bilateral hearing loss since 2009 was referred from the ENT department to the psychiatric OPD. He presented with a three-month history of hearing prayer chants and hymns. Initially, he believed these sounds were from his local temple playing them at unusual times. However, he soon realized the sounds persisted even when the temple was quiet and seemed to follow him throughout the day, including at work despite the loud machinery noises. As the hallucinations intensified, they caused him significant distress. He frequently checked with his family, asking if they could hear the chants as well. He was able to describe the chants and identify them as Shiva mantras, which he often played or chanted during worship. He described his hallucinations as hymns and chants of Shiva mantras, frequently heard and chanted during his prayer sessions. He noted that the sounds seemed to come from somewhere behind his head and were more intense in quiet spaces and when he was not wearing his hearing aid. He could not will them away, even if he tried, and sometimes noticed they occurred alongside actual prayer chants. He was able to distinguish the hallucinations from ambient sounds. These hallucinations significantly disrupted his sleep, work performance, and social interactions, as their intensity was higher in quiet environments. He did not experience hallucinations in other modalities (tactile, olfactory, and visual). He had no family history of psychiatric illness (schizophrenia, depression, OCD, and suicide) and no history of past psychiatric illness.

His medical history revealed long-standing bilateral chronic suppurative otitis media (CSOM) since 2009, for which he underwent tympanoplasty and mastoidectomy on his left ear and was prescribed a hearing aid in 2017. The patient was advised to undergo treatment for his right ear as well, but he was unable to do so due to financial constraints. He has a smoking habit and denies the use of other substances. He has no history of chronic systemic illnesses (diabetes and hypertension), head injury, seizures, and changes in mood. No changes in energy levels, appetite, or cognitive functioning.

On examination

The patient was conscious and oriented, with a blood pressure of 130/80 mmHg, pulse of 88/min, respiratory rate of 14/min, SpO_2_ of 98%, normal cardiovascular and respiratory exams, a soft, non-tender abdomen with normal bowel sounds, and no focal neurological deficits except for bilateral hearing loss. Pure tone audiometry revealed the right ear: 61.6 dBHL and the left ear: 58.3 dBHL.

Mental status examination

The patient was kempt, walked into the room, and sat on the chair offered; he exchanged greetings with the therapist. Rapport was established. During the psychiatric evaluation, the patient was cooperative and slightly anxious. He had normal speech; the interview was focused on his concerns about the chants he was hearing, and the difficulties he had concentrating because of their occurrence. He had MH. He did not have any intrusive, repetitive thoughts or compulsive actions. He did not have delusions. No thought withdrawal, thought insertion, or broadcasting. Mini mental status examination (MMSE) was performed; he obtained a score of 28 ruling out neurocognitive decline.

Complete blood count, complete metabolic panel, renal function test, and serum electrolyte levels were done and found to be within normal limits (Table 1).

**Table 1:** Blood Investigations and Results

Test	Value	Reference Range
Hematology		
Hemoglobin	15.7 g/dL	13.5-18 g/dL
Total WBC Count	7200 Cells/Cumm	4000-10000 Cells/Cumm
Differential Count		
Polymorphs	66%	40-70%
Lymphocytes	26%	20-45%
Eosinophils	4%	1-6%
Monocytes	4%	2-10%
Basophils	0%	0-1%
Platelet Count	290000 Cell/Cumm	140000-440000 Cells/Cumm
PCV	51.3	40-54%
Total RBC Count	6.00 mill/Cumm	4.5-6 mill/Cumm
Biochemistry		
Fasting Blood Sugar	95 mg/dL	70-115 mg/dL
Post Prandial Blood Sugar	127 mg/dL	80-140 mg/dL
Lipid Profile		
Cholesterol	173 mg/dL	120-200 mg/dL
Triglycerides	112 mg/dL	50-200 mg/dL
HDL	43 mg/dL	35-80 mg/dL
VLDL	22 mg/dL	5-40 mg/dL
Renal Function Test		
Urea	46 mg/dL	10-50 mg/dL
Creatinine	0.9 mg/dL	0.7-1.4 mg/dL
Serum Uric Acid	6.8 mg/dL	3.4-7 mg/dL
Serum Electrolytes		
Sodium	135 mEq/L	135-145 mEq/L
Potassium	3.6 mEq/L	3.5-5 mEq/L
Chloride	103 mEq/L	96-108 mEq/L
Bicarbonate	23 mEq/L	22-29 mEq/L

The patient was advised to undergo neuroimaging studies but he declined it due to financial constraints. A provisional diagnosis of ACBS was done.

The treatment plan incorporated both non-pharmaceutical and pharmaceutical approaches. The non-pharmaceutical approach focuses on education about the condition, reassurance, and encouraging consistent hearing aid use. Medications include a low dose of risperidone, an antipsychotic drug. The patient was started on 1 mg of risperidone, nightly dose, and gradually escalated to 2 mg after three days. The patient experienced significant improvement over two weeks; he reported reduced frequency and intensity of hallucinations. He discontinued medication after a month due to perceived symptom resolution but experienced a relapse. Risperidone was restarted with an emphasis on medication adherence. Follow-up visits show occasional, manageable hallucinations that did not interfere with daily life.

## Discussion

This case discussion explores the unclear etiology of MH, emphasizing its established link with hearing loss. It explores how auditory deprivation may lead the brain to generate perceived sounds. While there is no single cure for MH, a multi-pronged approach has proven effective. This includes non-pharmaceutical methods such as education, reassurance, hearing aid optimization, and activities like music therapy. Additionally, medications such as low-dose atypical antipsychotics, antiepileptics, and antidepressants have shown promise in some cases [7].

Our case report describes a rare instance of MH without psychiatric illness. A PubMed search using "auditory Charles Bonnet syndrome" and "Oliver Sacks syndrome" yielded 38 articles, highlighting the need for further exploration. Currently, there are no definitive management strategies for ACBS, but both non-pharmacological and pharmacological approaches are utilized.

Several case studies illustrate the diverse presentations of MH in individuals with ACBS. For example, an 81-year-old woman reported hearing hymns, influenced by her religious upbringing, without other voices or visual hallucinations. Neither she nor her family were concerned about her memory or mood. She experienced frontal headaches, occasional dizziness, and long-term hearing issues requiring hearing aids [8]. Managing MH in ACBS often involves addressing the underlying hearing loss. Hearing aids or cochlear implants can restore auditory input and alleviate hallucinations. Pharmacological treatments like antipsychotics, antidepressants, and antiepileptics may be considered if the hallucinations are distressing or impair the quality of life [9].

A study conducted by Sakimoto et al. described 6 patients who were effectively treated with carbamazepine and clonazepam. In the study, the use of antipsychotics was found to be ineffective [10]. Another study by Charlotte Kukstas emphasized the importance of treating the underlying cause and also listed medications known to trigger MH which include olanzapine, clomipramine, carbamazepine, valproate, and donepezil [11]. A case series by Nattakarn Limphaibool described six cases with a history of cerebrovascular disease presenting with MH. The patients showed improvement after being treated with carbamazepine [12].

We employed a combined approach in our case supported by several reports that describe successful management using these strategies. This report highlights the successful reduction of symptoms with risperidone and emphasizes the role of sensory deprivation in the development of hallucinations. A limitation of this study is the lack of brain imaging and EEG.

## Conclusions

This case report presented a 51-year-old male with a history of hearing loss who developed MH. The case highlights the potential link between hearing loss and the emergence of these non-psychotic auditory experiences. Initially mistaking the persistent prayer songs for external sounds, the patient eventually recognized them as hallucinations. Notably, the frequency of these hallucinations decreased when he used his hearing aid, suggesting a role for sensory deprivation in their development.

Following a diagnosis of ACBS, a treatment plan combining medication and non-pharmacological interventions was implemented. Risperidone, an antipsychotic medication, effectively reduced the frequency and intensity of the hallucinations, leading to improved sleep and concentration. However, the patient's initial reluctance to seek help due to the stigma surrounding mental health and his subsequent discontinuation of medication upon symptom relief demonstrates the importance of addressing these concerns. Open communication and education about the condition were crucial in encouraging medication adherence and ensuring long-term management.

This case underscores the multifaceted approach necessary for managing MH. Addressing hearing loss through hearing aids can be a valuable tool. Additionally, a combination of medication and patient education can empower individuals to cope with these experiences and maintain their quality of life. While further research is needed to fully understand the underlying mechanisms of MH, this case report offers valuable insights for clinicians managing patients with similar presentations.
